# Malaria infection and predictor factors among Chadian nomads’ children

**DOI:** 10.1186/s12889-024-18454-5

**Published:** 2024-03-28

**Authors:** Azoukalné Moukénet, Kebfene Moudiné, Ngarkodje Ngarasta, Clement Kerah Hinzoumbe, Ibrahima Seck

**Affiliations:** 1 Cheikh Anta Diop University, Dakar, Senegal; 2 University of Ndjamena, Ndjamena, Chad; 3 Abomey-Calavi University, Abomey-Calavi, Benin; 4 UNDP, Ndjamena, Chad

**Keywords:** *Plasmodium*, Prevalence, Infection, Long-lasting insecticidal nets, Seasonal malaria chemoprevention, Factors, Children, Nomads, Chad, Malaria

## Abstract

**Background:**

In Chad, malaria remains a significant public health concern, particularly among nomadic populations. Geographical factors and the mobility of human populations have shown to be associated with the diversity of *Plasmodium* species. The study aims to describe the malaria prevalence among nomadic children and to investigate its associated factors.

**Methods:**

A cross-sectional study was conducted in February and October 2021 among nomadic communities in Chad. Blood sample were collected and tested from 187 Arab, Fulani and Dazagada nomadic children aged 3–59 months using malaria rapid diagnostic test (RDT). A structured electronic questionnaire was administered to their parents to collect information about the socio‑economic data. Malaria testing results were categorized according to the SD BIOLINE Malaria Ag Pf/Pan RDT procedures. Logistic regression analysis was used to determine key risk factors explaining the prevalence of malaria. STATA version IC 13 was used for statistical analysis.

**Results:**

The overall malaria prevalence in nomadic children was 24.60%, with 65.20% being *Plasmodium falciparum* species and 34.8% mixed species. Boys were twice as likely (COR = 1.83; 95% CI, 0.92–3.62; *p* = 0.083) to have malaria than girls. Children whose parents used to seek traditional drugs were five times more likely (AOR = 5.59; 95% CI, 1.40–22.30, *p* = 0.015) to have malaria than children whose parents used to seek health facilities. Children whose parents reported spending the last night under a mosquito net were one-fifth as likely (AOR = 0.17; 95% CI, 0.03–0.90, *p* = 0.037) to have malaria compared to children whose parents did not used a mosquito net. Furthermore, Daza children were seventeen times (1/0.06) less likely (AOR = 0.06; 95% CI, 0.01–0.70, *p* = 0.024) to have malaria than Fulani children and children from households piped water as the main source were seven times more likely (AOR = 7.05; 95% CI, 1.69–29.45; *p* = 0.007) to have malaria than those using surface water.

**Conclusions:**

Malaria remains a significant public health issue in the nomadic communities of Chad. Community education and sensitization programs within nomad communities are recommended to raise awareness about malaria transmission and control methods, particularly among those living in remote rural areas. The National Malaria Control Program (NMCP) should increase both the coverage and use of long-lasting insecticidal nets (LLINs) and seasonal malaria chemoprevention (SMC) in addition to promoting treatment-seeking behaviors in nomadic communities.

**Supplementary Information:**

The online version contains supplementary material available at 10.1186/s12889-024-18454-5.

## Background

Malaria is a public health disease caused by parasites belonging to the *Plasmodium* genus and transmitted to humans through the bites of infected female Anopheles mosquitoes that breed in aquatic habitats [1, 2]. According to the World Health Organization (WHO), an estimated 247 million cases of malaria worldwide in 2021, resulted in 619, 000 deaths. The WHO African Region bears a disproportionately high share of the global malaria burden with 95% of malaria cases and 96% of malaria deaths. Children under 5 accounted for about 80% of all malaria deaths in the Region [3]. In Chad, malaria is endemic with areas at risk of epidemics. In 2021, the country recorded around 3.5 million cases of malaria and 11, 744 deaths [3]. Malaria has consistently been the primary health problem reported in health facilities in Chad, with around 50% of malaria cases reported in children under five years old [4, 5]. Overall, three *Plasmodium* species (*falciparum, malariae* and *ovale*) are incriminated, but 98% of malaria cases are attributable to *P. falciparum* [6, 7].

Well-explored risk factors for malaria infection include biological factors such as age (children under 5), gender [8, 9], malnutrition status of children [10], socioeconomic factors such as poverty [11], poor awareness and knowledge about malaria prevention and control [12]. Exposure to mosquito breeding sites [11–13] increases the risk of malaria infection while the use of preventive interventions such as a new LLIN [14–17] reduces this risk. Regarding the diversity of malaria parasites, geographical proximity to areas with various *Plasmodium* species and the movement of human populations particularly in malaria-endemic areas for a long period have been associated with *P. falciparum* and *P. vivax* [18]..

The Chadian population consists of both mobile and settled populations with around 3.5% being nomadic [19]. Nomads mainly inhabit the areas between the Saharan and Sahelian zones. Changes in climate [20], economy [21] and politics [22] over the past decades have led to a considerable extension of pastoral mobility toward the Sudanian areas [23]. Nomads traverse various malaria transmission areas within the country Chad [23] and sometimes cross the borders in search of pasture and water for their herds. As asymptomatic carriers of malaria parasites, these populations constitute a reservoir of *Plasmodium* species from other countries.

Numerous malaria control interventions worldwide have been implemented and proven effective [24–35]. The combination of some interventions such as the long-lasting insecticide-treated net (LLIN) and the seasonal malaria chemoprevention (SMC) has demonstrated increased effectiveness [27]. Chad has adopted SMC with sulphadoxine-pyrimethamine and amodiaquine (SPAQ) to prevent seasonal malaria among children aged 3–59 months in the Sahel region [36]. There is promotion of the use of LLINs distributed through mass distribution and routine basis [37]. The last mass LLIN distribution campaign in Chad occurred in 2023.

Despite deep understanding of malaria predictive factors, most studies have focused on single factors, thus not providing a holistic recognition of the factors associated with malaria among nomadic communities. Both factors at the individual/household and community levels have been omitted. In Chad, few studies related to malaria in nomadic communities are available. Some of them have focused on knowledge and attitudes toward malaria [38], and coverage of control interventions among this community [37]. To address this gap, the objective of this study is to assess the prevalence of malaria and investigate the associated factors among nomadic communities in Chad.

## Participants and methods

### Study area and design

This cross-sectional study conducted in February and October 2021 among nomadic communities in the provinces of Hadjer Lamis and Chari Baguirmi in the Sahelian zone of Chad and in Moyen Chari in the Sudanian zone. In comparison to the national level (40.9%), the prevalence of malaria was moderate in Hadjer Lamis (15.9%), high in Chari Baguirmi (37.2%) and very high (68.1%) in Moyen Chari [39]. In addition to intermittent preventive treatment for pregnant women (IPTp), routine and mass LLIN distribution campaigns have been implemented in all three provinces whereas SMC is being implemented in Hadjer Lamis and Chari Baguirmi provinces targeting children aged 3–59 months.

Each year, transhumant nomads migrate in the endemic area from the Sahelian zone to the Sudanian zone and subsequently return to the northern part of the country with low or no malaria transmission, when the rainy season arrives. The structure of nomad habitat has been described elsewhere [38]. Accordingly, blood samples were taken twice a year from children aged 3–59 months, and none of these children have been sampled twice. The first samples were taken when the nomads were moving to the south (January– March) and the second set during the high malaria transmission period (July– October) when they reached the Sahelian zone.

### Study population

Nomadic children, specifically Arab or Fulani or Dazagada (Daza) children aged 3–59 months at the time of the study whose parents provided consent to blood sample collection were included. To mitigate the inflation of malaria prevalence due to parents selectively designating sick children, in each randomly selected household within the camps, a blood sample was obtained from the oldest child aged 3 to 59 months. If the result from the RDT was positive for *Plasmodium*, the child received a dose of antimalarial in accordance with the national treatment protocol [40].

### Sampling and recruitment

Within each of three nomad group a multi-stage cluster random sampling technique was employed, with the camp as first stage and the household as second-stage leading to a minimum sample of 180 study subjects. A provision of 4% was added to account for non-response. At the first level, from the list of camps provided by leaders of each three nomad groups, 51 camps were randomly selected using a random number draw. At the second stage, within each selected camp, surveyors used random number draws to select two households (four households for very large camps). For selected household, one member aged older than 18 years was requested for interviews and responded on behalf of the household to which he or she belongs. Additionally, the oldest child of the group of 3–59 months was chosen for blood sampling. Of the 187 children, 81 were blood sampled in January– March and 106 in July– October.

### Data collection

After being informed, the parents of the children were required to provide written consent, they indicated their agreement by affixing their signature or fingerprint on two copies of the cards. The demographic characteristics of the child (age and sex), facial temperature by thermoflash for some children and the results of the RDT (SD BIOLINE Malaria Ag Pf/Pan, Standard Diagnostics USA) were entered on the collection sheet with a unique and anonymous identification code for each child.

To record information on the parents’ sociodemographic characteristics, their knowledge and experiences regarding malaria, and their use of mosquito nets, a structured electronic questionnaire developed based on Peto et al. [37, 41] was administered to an adult who responded on behalf of the household during blood sample collection. The survey questionnaire was administered in February and October 2021 by three trained data collectors fluent in the local languages and experienced in collecting data for nomad immunization programs. The questionnaire included items on respondents’ socio-demographic characteristics; their knowledge and experiences regarding malaria; and their use of mosquito nets. The questionnaire was implemented in KoBoCollect v2021.2.4 [42], and was administered offline before responses were uploaded to the server once the WiFi connection was available. While data collectors were administering the questionnaire, health agents were collecting blood sample.

### Response variable

The results of malaria test were categorized as either “negative” or “positive” according to the SD BIOLINE Malaria Ag Pf/Pan RDT procedures. The SD Bioline^™^ Malaria Ag Pf/Pan test is a rapid, qualitative, and differential test designed to detection of the histidinerich protein 2 (HRP2) antigen of *Plasmodium falciparum* and the common *Plasmodium* lactase dehydrogenase (pLDH) of *Plasmodium* species in human whole blood. The reliability of RDT-based diagnosis is reported elsewhere [43–47].

### Explanatory variables

We evaluated the association between malaria prevalence and socioeconomic and contextual factors. The questionnaire included a variety of questions related to socioeconomic and contextual factors, listed below:

- characteristics of the individual households and children;
- contextual characteristics;
- and district and zone variables.

### Characteristics of the individual households and children

The main explanatory variables were: (i) the child’s temperature; (ii) the child’s age (3–59 months); (iii) the gender of the child and household characteristics such as (iv) household wealth quintiles, categorized as “lowest”, “middle” and “highest” economic levels; (v) ethnic group (Arab, Dazagada and Fulani); (vi) marital status of the head of household categorized as, “widowed/divorced”, “monogamy” and “polygamy”; (vii) reflex in case of malaria, including seeking a “street drug seller”, “health facility”, “traditional drug”; and (viii) utilization of LLIN and knowledge of malaria.

Common principles used to measure knowledge of malaria include questions on transmission and preventive interventions [48]. This study used similar principles published elsewhere [37]. Dimensions included were related to the periods of high transmission (rainy season), the group most at risk (children and pregnant women), means of transmission (mosquito bite) and common symptoms (fever, chills, muscle pain, stomach pain, diarrhea, nausea and vomiting). The dimension of interventions related to sleeping under a LLIN as mean of protection against malaria. Each correct response to question was scored one point and zero for wrong answers. An overall knowledge score was calculated by summing the scores for each respondent across all questions. Those with scores of 2.5 (mid-point between 0 and 5) or above were considered to have good knowledge, while those with lower scores were categorized as having poor knowledge about malaria.

### Contextual characteristics

These characteristics were: ix) place of residence categorized as, “urban” or “rural”. Urban residence includes townships, municipalities and cities; x) the season; xi) the malaria control intervention in place (LLINS and SMC).

### District and zone variables

These included: viii) all 3 administrative provinces; and ix) all district areas.

## Laboratory methods

### Rapid diagnostic test

Capillary blood samples were collected by study staff using a finger prick. One drop of blood was used to perform a malaria RDT (SD BIOLINE Malaria Ag PF/Pan, Standard Diagnostics USA). Blood samples were collected by three trained health agents who had previously collected blood sample and perform RDT during the National Malaria Survey (ENIPT) [39], in addition to participating in nomad immunization programs. Health agents were responsible for collecting children’s blood samples and performing RDT, while data collectors were administered questionnaires to parents. The RDT kits used in this study were obtained from health districts as recommended by the Ministry of health (MOH) and used in public health facilities.

## Statistical methods

The data collected during each period were entered into Excel files, and STATA version IC 13 was used for statistical analysis. Percentages, means and standard deviations were calculated. Differences in proportions were assessed with exact Fisher’s tests. Sample means were compared by unpaired Student’s t tests. Values of *p* < 0.05 were considered significant. The parasitaemia rate was defined as the percentage of children with positive results from RDTs among the total of children surveyed. Few children had their temperature taken; therefore, the malaria prevalence rate was not processed, although children carrying *Plasmodium* were considered to have malaria.

Principal Component Analysis (PCA) [49] was used to develop wealth categories for the households based on access to facility including potable water and ownership of durable assets including solar kit, radio, telephone, cart tracked by animal, motorcycle/scooter, and caws/camels and sheep/goats per capita. Access and ownership were coded as 0 or 1 and missing cases were excluded. The first dimension of the PCA was taken as the household wealth score and range into tertiles; households were then placed into socioeconomic categories based on their scores.

We performed a descriptive analysis and presented participants’ social and demographic characteristics stratified by RDT results. We then employed exact Fisher’s tests to assess any difference in malaria prevalence by socioeconomic and contextual factors. Logistic regression analysis was conducted to identify the factors associated with malaria prevalence among nomads in Chad. Crude (COR) and adjusted odds ratios (AOR) were calculated to check statistical associations between the dependent and independent variables using the binary logistic regression and multivariable logistic regression models. All variables in the study were initially tested for association with malaria prevalence using a binary logistic regression model. Those showing a significant statistical association (*p* < 0.05) were added into the multivariable analysis model to assess whether the association persisted after controlling against all other variables. A 95% confidence intervals and the 5% significance level were calculated for all odds ratios.

## Results

### Characteristics of the study population

**Fig. 1:**
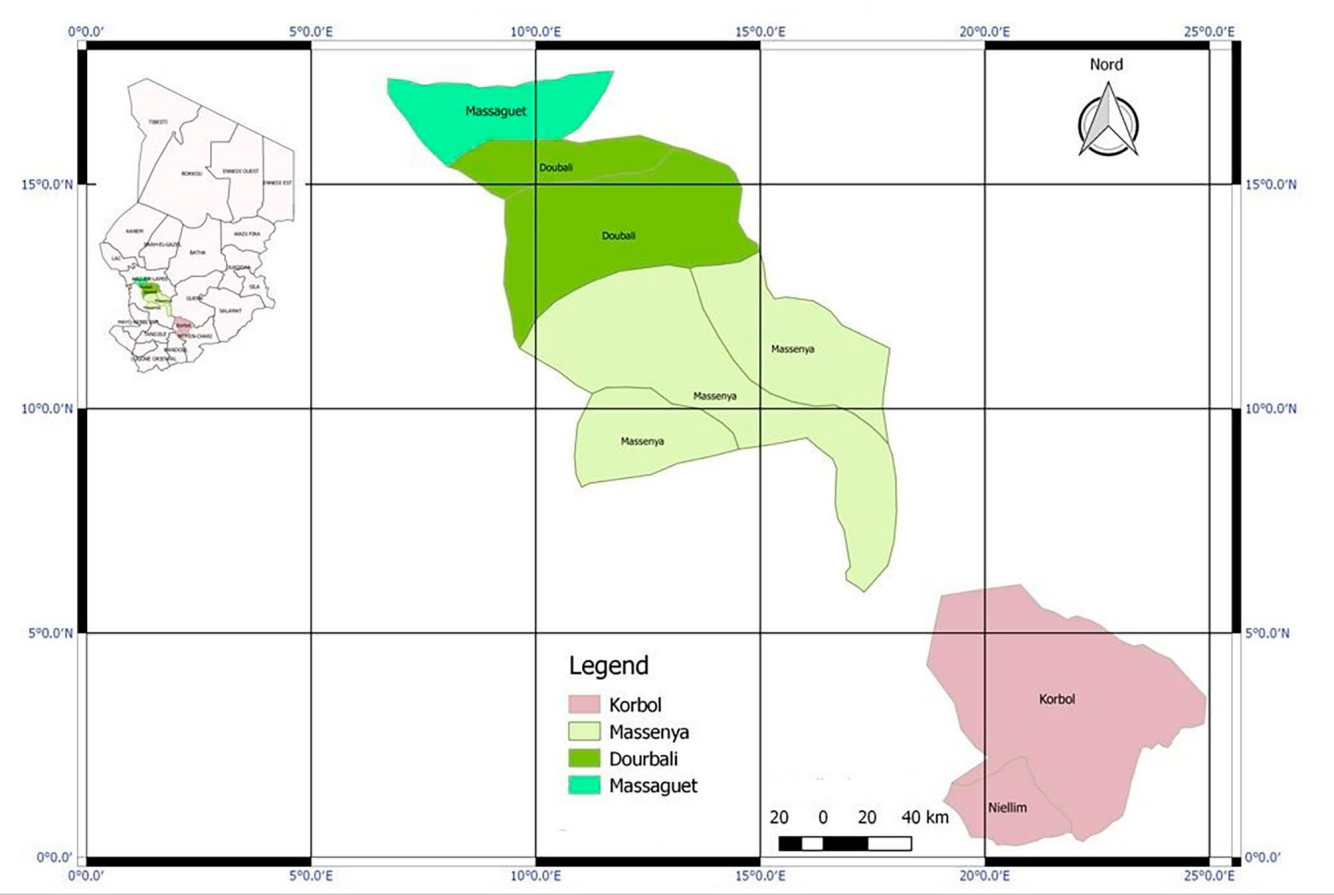
Map of study area

Overall, 187 children aged 3–59 months were enrolled in this study, distributed across the following districts and provinces: Dourbali (17.6%) and Massenya (13.9%) in Chari Baguirmi province (31.5%); Massaguet (55.1%) in the Hadjer Lamis province (55.1%) and Niellim (13.4%) in Moyen Chari province (13.4%) were enrolled in this study (Table 1; Fig. 1). Of them, 90 (48.1%) were female, and the enrolled children belonged to Arab (36.4%), Daza (36.4%) and Fulani (27.2%) ethnic groups. Concerning malaria, 86 (46.0%) and 36 (19.2%) participants came from households using street drugs and traditional drugs respectively in case of malaria episodes. Additionally, 154 children (82.4%) were from households with poor utilization of LLINs, and 162 (86.6%) of the participants were living in SMC areas (Table 1).

**Table 1:** Characteristics of household and children and crude results of malaria test (*n* = 187)

Variables	N (%)	Malaria test n (%)		*p*
		Negative 141 (75.40)	Positive 46 (24.60)	
Gender				
Female	90 (48.1)	73 (81.1)	17 (18.9)	0.091
Male	97 (51.9)	68 (70.1)	29 (29.9)	
Age - range in months				
3–12	19 (10.2)	13 (68.4)	6 (31.6)	0.574
12–60	168 (89.8)	128 (79.2)	40 (23.8)	
Household Wealth Quintiles				
Lowest	80 (42.8)	68 (85.0)	12 (15.0)	0.020
Middle	44 (23.5)	32 (72.7)	12 (27.3)	
Highest	63 (33.7)	41 (65.1)	22 (34.9)	
Ethnic group				
Arab	68 (36.4)	53 (77.9)	15 (22.1)	0.004
Daza	68 (36.4)	58 (85.3)	10 (14.7)	
Fulani	51 (27.2)	30 (58.8)	21 (41.2)	
Head of household marital status				
Widowed/divorced	13 (7.0)	9 (69.2)	4 (30.8)	0.034
Monogamy	136 (72.7)	109 (80.1)	27 (19.9)	
Polygamy	38 (20.3)	23 (60.5)	15 (39.5)	
Reflex in case of malaria				
Street drug seller	86 (46.0)	71 (82.6)	15 (17.4)	0.091
Health facility	65 (34.8)	44 (67.7)	21 (32.3)	
Traditional drug	36 (19.2)	26 (72.2)	10 (27.8)	
Knowledge of malaria				
Poor	40 (76.9)	12 (23.1)	52(27.8)	0.851
Accurate	101 (74.8)	34 (25.2)	135 (72.2)	
Utilization of mosquito net (all type)				
Poor	26 (76.5)	8 (23.5)	34 (18.3)	1.000
Accurate	115 (75.7)	37 (24.3)	152 (81.7)	
Utilization of LLIN				
Poor	154 (82.4)	116 (75.3)	38 (24.7)	1.000
Accurate	33 (17.6)	25 (75.8)	8 (24.2)	
Main water source				
Surface	46 (71.9)	18 (28.1)	64 (34.2)	0.250
Piped	35 (68.6)	16 (31.4)	51 (27.3)	
Tower	8 (88.9)	1 (11.1)	9 (4.8)	
Well	52 (82.5)	11 (17.5)	63 (33.7)	
Province				
Chari Baguirmi	59 (31.5)	40 (67.8)	19 (32.2)	0.001
Hadjer Lamis	103 (55.1)	90 (87.4)	13 (12.6)	
Moyen Chari	25 (13.4)	11 (44.0)	14 (56.0)	
District				
Dourbali	33 (17.6)	21 (63.6)	12 (36.4)	0.00003
Massaguet	103 (55.1)	90 (87.4)	13 (12.6)	
Massenya	26 (13.9)	19 (73.1)	7 (26.9)	
Niellim	25 (13.4)	11 (44.0)	14 (56.0)	
SMC area				
Yes	162 (86.6)	130 (80.2)	32 (19.8)	0.0003
No	25 (13.4)	11 (44.0)	14 (56.0)	
Season				
Rainy	106 (56.7)	83 (78.3)	23 (21.7)	0.308
Dry	81 (43.3)	58 (71.6)	23 (28.4)	

### Malaria prevalence according to characteristics of the study participants

**Fig. 2:**
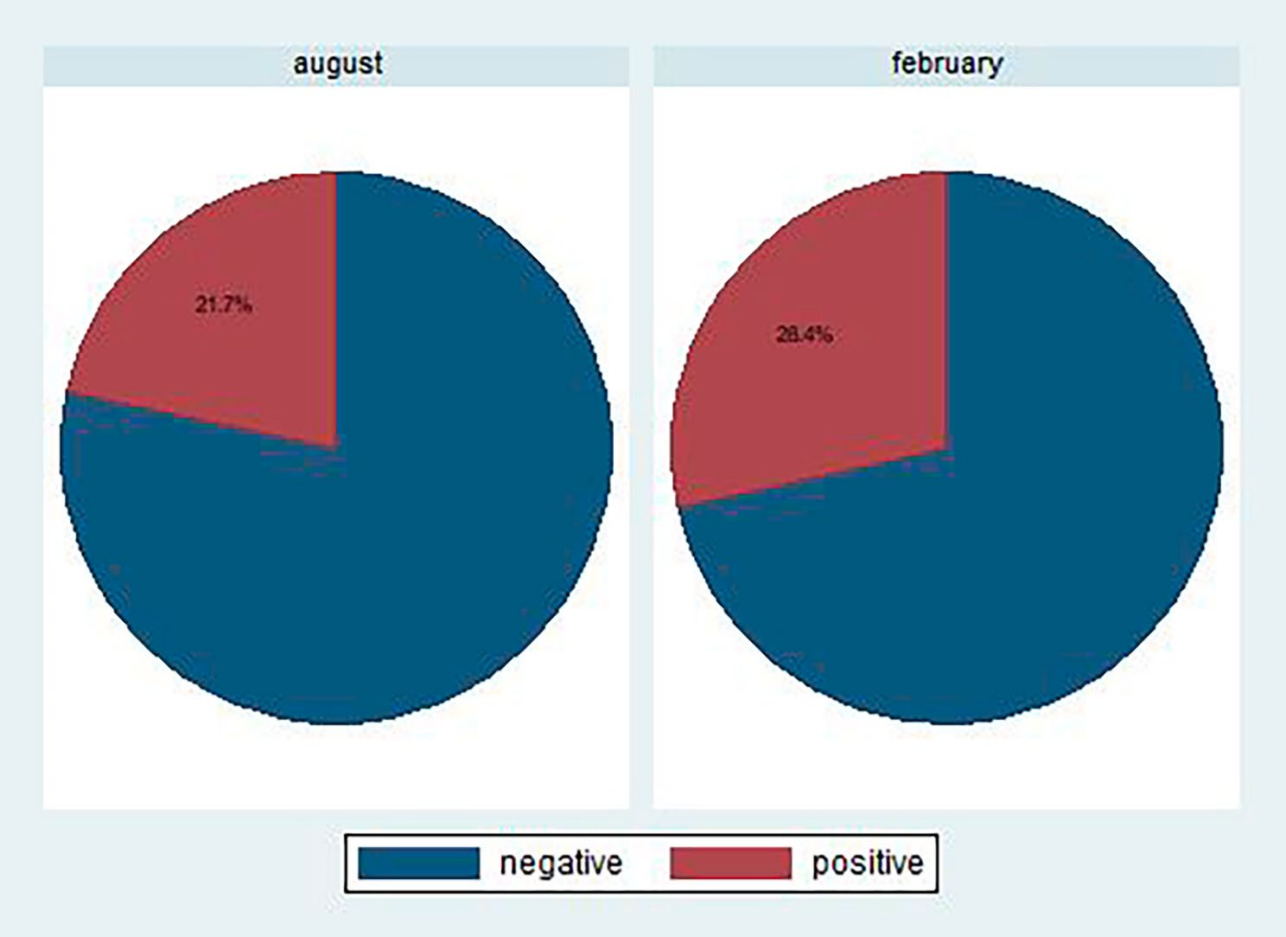
Malaria prevalence by month of data collection

Overall 46 (24.60%) children were tested positive for malaria. The malaria prevalence was 21.7% and 28.4% respectively for blood sample collected in October and February 2021 (Fig. 2). Fisher’s exact test revealed a notably higher prevalence of malaria in children from the southern regions of the Sahelian zone, specifically Massenya and Dourbali, as well as in Niellim located in the south of the country, compared to the northern part of Sahelian counterparts in Massaguet. Specifically malaria positivity rates were 26.9% in Massenya, 36.4% in Dourbali and 56.0% in Niellim, significantly higher than the 12.6% observed in Massaguet. A significantly higher proportion of participants were tested positive in the area not receiving SMC (56.0%). Regarding the individual characteristics of participants and that of their households, a significant proportion of positive tests were found in boys (29.9%) compared to girls (18.9%). Additionally, positive test results were significantly higher among children from the Fulani (41.2%) and Arab (22.1%) ethnic groups than among those from the Daza (14.7%) ethnic group. A significantly larger proportion of positive tests was also found among children from households seeking traditional drugs (27.8%) and health facilities (32.3%) compared to street drug sellers (17.4%) in the case of malaria episodes (Table 1).

### Malaria prevalence according to Plasmodium species

The malaria RDT results according to *Plasmodium* species are presented in Table 2. Out of 46 positive malaria tests, 16 (34.8% of positive test results) exhibited mixed species (*Plasmodium falciparum, Plasmodium ovale, Plasmodium malaria and/or Plasmodium vivax*). Mixed *Plasmodium* species were more frequently found in Daza children (70.0%), followed by Fulani children (66.7%). Concerning the use of mosquito nets, mixed *Plasmodium* species were identified in children whose parents reported not spending the last night under mosquito nets.

**Table 2:** Typology of *plasmodium* found in the study, n (%)

Variables	P. falciparum 30 (65.2)	Mixed species 16 (34.8)	*P*
Gender			
Female	12 (81.1)	5 (18.9)	0.750
Male	18 (62.1)	11 (37.9)	
Age - range in months			
3–12	3 (0.0)	3 (100.0)	0.405
12–60	27 (67.5)	13 (32.5)	
Ethnic group			
Arab	13 (86.7)	2 (13.3)	0.020
Daza	3 (30.0)	7 (70.0)	
Fulani	14 (66.7)	7 (33.3)	
Reflex in case of malaria			
Street drug seller	8 (53.3)	7 (46.7)	0.163
Health facility	13 (61.9)	8 (38.1)	
Traditional drug	9 (90.0)	1 (10.0)	
Utilization of mosquito net (all type)			
Poor	1 (12.5)	7 (87.5)	0.001
Accurate	29 (78.4)	8 (21.6)	
Province			
Chari Baguirmi	6 (85.7)	1 (14.3)	0.529
Hadjer Lamis	16 (64.0)	9 (36.0)	
Moyen Chari	8 (57.1)	6 (42.9)	
District			
Dourbali	10 (83.3)	2 (16.7)	0.158
Massaguet	6 (46.2)	7 (53.8)	
Massenya	6 (85.7)	1 (14.3)	
Niellim	8 (57.1)	6 (42.9)	

### Factors associated with a positive test for malaria prevalence

Among all variables, geographic characteristics such as province, SMC area and individual or household characteristics like gender, ethnicity, socioeconomic status and the household’s behavior during malaria episodes, were significantly associated with malaria positivity (*p* < 0.05) using the crude logistic regression (Table 3). Additionally, the use of mosquito nets (all types and LLINs), knowledge of malaria and the main source of water used by the household were included in the logistic regression adjusting for all other variables and only those significantly associated with malaria were retained.

After adjusting for other individual, household and geographic characteristics, Daza children were seventeen times less likely (AOR = 0.06; 95% CI, 0.01–0.79, *p* = 0.024) to have malaria than Fulani children. Children whose parents used to seek traditional drugs were five times more likely (AOR = 5.48; 95% CI, 1.38–21.72, *p* = 0.016) to have malaria than children whose parents used to seek health facilities. Children whose parents reported spending the last night under a mosquito net were one fifth as likely (AOR = 0.17; 95% CI, 0.03–0.93, *p* = 0.041) to have malaria compared to children whose parents did not spend the last night under a mosquito net. Children from households with piped water as main source were seven times more likely (AOR = 7.05; 95% CI, 1.69–29.45; *p* = 0.007) to have malaria than children from households using surface water. Children from areas not implementing SMC were twenty times more likely (AOR = 20.61; 95% CI, 2.91–145.77; *p* = 0.002) to have malaria than children from areas implementing SMC.

**Table 3:** Factors associated with malaria positivity among nomads

Categories	COR (95% CI)	*p*	AOR (95% CI)	*p*
Ethnic group				
Fulani (ref)	1		1	
Daza	0.25 (0.10–0.59)	0.002	0.06 (0.01–0.70)	0.024
Arab	0.40 (0.18–0.90)	0.026	0.72 (0.22–2.35)	0.587
Reflex in case of malaria				
Health facility (ref)	1		1	
Street drug seller	0.44 (0.21–0.95)	0.036	1.56 (0.45–5.45)	0.487
Traditional drug	0.81 (0.33–1.97)	0.637	5.48 (1.38–21.72)	0.016
Utilization of mosquito net (all type)				
Poor (ref)	1		1	
Accurate	1.05 (0.44–2.51)	0.920	0.17 (0.03–0.93)	0.041
Main water source				
Surface (ref)	1		1	
Piped	1.17 (0.52–2.61)	0.705	7.05 (1.69–29.45)	0.007
Tower	0.32 (0.04–2.74)	0.298	2.18 (0.18–26.89)	0.543
Well	0.54 (0.23–1.26)	0.155	9.02 (0.94–86.63)	0.057
SMC area				
Yes (ref)	1		1	
No	5.17 (2.15–12.46)	0.0003	20.61 (2.91–145.77)	0.002
Province				
Chari Baguirmi (ref)	1		NA	NA
Hadjer Lamis	0.61 (0.23–1.61)	0.320		
Moyen Chari	3.45 (1.07–11.16)	0.038		
Gender				
Female (ref)	1		NA	NA
Male	1.83 (0.92–3.62)	0.083		
Household Wealth Quintiles				
Lowest (ref)	1		NA	NA
Middle	2.13 (0.86–5.25)	0.102		
Highest	3.04 (1.36–6.79)	0.007		
Knowledge of malaria				
Poor (ref)	1		NA	NA
Accurate	1.12 (0.53–2.38)	0.764		
Utilization of LLIN				
Poor (ref)	1		NA	NA
Accurate	0.98 (0.41–2.35)	0.958		

Note: CI = 95% Confidence Interval, NA = Not applicable (not retained in the model), COR = Crude Odd Ratio, AOR = Adjusted Odd ratio

## Discussion

Malaria remains a public health challenge in sub-Saharan Africa, including Chad despite ongoing efforts for control and elimination through various interventions and financial investments. It is difficult to reach mobile communities that are more exposed to malaria than the general population. This study aimed to assess the prevalence of malaria and explore individual/household and community factors associated with malaria among nomadic communities in Chad.

This study highlighted the burden of malaria in Chad, with a national prevalence in the general population of 40.9% with high variability according to the endemicity area. The prevalence of malaria is moderate in Hadjer Lamis (15.9%), high in Chari Baguirmi (37.2%) and very high (68.1%) in Moyen Chari [39]. In this study, the prevalence of malaria was 24.6% which is low compared to the aforementioned study conducted in the general population without specificity for nomad considerations. However, this result is consistent with other studies conducted in the nomadic setting of Chad reporting a malaria prevalence of up to 30% [50]. The high prevalence documented in this study is also in line with studies in other African countries among nomadic populations such as Fulani pastoralists in southwestern Nigeria, recording a prevalence of 33.6% [51]. Additionally, in the general population of Ghana, a prevalence of 20.9% was reported [9].

Among malaria positive tests, 34.8% were found to be a mix of malaria parasites. This result underscores the necessity for further analysis to assess the malaria parasite species in Chad. Studies forming the basis for malaria management protocols in Chad tend to be out dated, reporting 98% of species as *Plasmodium falciparum* and only 2% as other malaria parasite species [6]. Furthermore, the results from this study indicate a high percentage of mixed *Plasmodium* species in Daza children, followed by Fulani children and children whose parents are not accurate users of mosquito nets. This result can be explained by the trajectory followed by both the Daza and Fulani groups during transhumance, with Daza group crossing the country in the east and the Fulani group in the south.

The current study revealed significant variation in the odds of malaria prevalence across provinces. In Chad, approximately 53% of the total land has climatic conditions favorable for malaria transmission (sahelian and sudanian area), covering 98% of the population. Malaria transmission in the Sahelian area spans between 3 and 4 months seasonally, while the Sudanian exhibits high endemicity throughout the year. Other factors, including altitude, temperature, humidity, rainfall, presence of breeding sites, and agricultural activities within provinces may explain variation in the prevalence of malaria between provinces. In this study, the higher malaria prevalence in Moyen Chari (Sudanian area) compared to Chari Baguirmi (Sahelian area) can also be attributed to the impact of malaria intervention in the Sahelian area (SMC) in contrast to the Sudanian area. Therefore, the SMC area was found to be associated with a lower prevalence of malaria.

In the current study, male participants were more likely to test positive for malaria than female. This finding aligns with the results of a study from Ghana and Cameroon establishing a significant association between gender and malaria prevalence, with males having a higher prevalence than females [8, 9, 12]. This could be explained by nomadic boys assisting parents with herds in the early evenings and, therefore, exposing them more to mosquito vectors. Additionally, other studies have suggested that boys tend to play outdoors in the early evenings more frequently than girls, resulting in increased exposure to mosquito vectors [8].

The study’s findings indicated a higher risk for malaria prevalence for Fulani children compared to Daza children. This result can be attributed to the area of exposure visited by these nomad groups. Fulani nomads ventured further into the Sudanian Chad than Daza who mostly stayed in the Sahelian zone. Furthermore, in recent decades, due to climatic, economic and political changes, a significant increase in pastoral mobility has been recorded in the Sudanian zone [23, 38]. Moreover, some nomadic groups, such as the Fulani, have started transitioning to a more sedentary lifestyle in the Sudanese area [38].

Children whose parents sought traditional drugs for malaria treatment were more likely to have malaria than those whose parents sought health facilities. This finding can be explained by the low efficacy of traditional drugs against malaria and the lack of awareness regarding malaria prevention and treatment. Since these drugs often lack active principles to cure malaria, children may continue to harbor malaria parasites. As indicated elsewhere [38], traditional drugs in nomadic communities sometimes include ‘koulkul tree leaves’, ‘camel urine’ and ‘beef urine’ or ‘milk butter’. However, these results underscore the imperative need to enhance the availability and accessibility of health services. It has been mentioned elsewhere that the cost of health care and the severity of illness were the primary reasons for selecting health services in nomadic communities [38]. Furthermore, such interventions should be complemented by health promotion activities, as these factors influence community treatment-seeking patterns and contribute to ongoing malaria transmission.

Among all participants in the study, four to five mentioned spending the last night under a mosquito net of various types. While some may use the mosquito nets to protect themselves from other insect bites [38], the use of mosquito nets has proven effective in protecting children from malaria. Children whose parents reported spending the last night under a mosquito net were less likely to have malaria than those whose parents had not used a mosquito net. This result underscores the high potential for preventing malaria within this community by utilizing mosquito nets. This impact might have been more significant if nomads had access to LLINs; however, as mentioned elsewhere [37, 38], this community has limited access to LLINs.

Children from households with piped water as main source of water were more likely to have malaria than those from households relying on surface water. Typically, piped water and wells are in proximity to nomad camps, enabling women and children to fetch water late at night. In contrast, surface water is far from camps, requiring women and children to seek water during the day with less exposure to mosquito bites. Additionally, queuing for other water sources may be longer than for surface water; resulting in a shorter duration of exposure to mosquito bites. This result appears contrary to findings in Tanzania [11], which considered piped water as free from stagnant water. However, in the nomad camps, all water sources are used by both humans and animals, meaning stagnant water surrounds all water sources. These stagnant waters serve as breeding ground mosquitoes, facilitating the development of the larvae into adult mosquitoes.

### Strengths and limitations

This study has some limitations, including a small size of enrolled children, which may affect its representativeness for the entire nomadic child population. Nevertheless, the results provide insights into the overall descriptive situation of nomadic children. There may be potential bias in measuring LLIN use among children tested for malaria as only member per household responds on behalf of the household without specifying if children were under the mosquito net. Another limitation of this study was the reliance on a cross-sectional survey conducted at the end of the rainy season and the dry season when mosquito density and malaria transmission may be lower than in the rainy season. The study could not delve into parasite typology research through polymerase chain reaction (PCR) analysis due to funding. In terms of recommendations, interventions would be more cost-effective if tailored to the district level rather than at the provincial or national level. However, the study can be valuable for understanding factors associated with an increased likelihood of malaria positivity, guiding stakeholders in the implementation of malaria prevention, control and elimination strategies in nomadic communities, and the entire population in Chad. Furthermore, the study suggests the need for additional research to assess the typology of malaria parasites.

## Conclusions

The study findings revealed a malaria prevalence of 24.6% with 34.8% of positive test indicating a mix of malaria parasites. This prevalence is relatively lower as compared to other studies conducted in other settings. Factors influencing malaria infection in nomad communities include the participants’ ethnic group, the reflex of households in malaria cases, the utilization of mosquito nets regardless of type, the main water source used by households and the participants’ living area. The findings underscore that malaria remains a public health concern in Chad, particularly in nomadic communities. The gathered information can guide the implementation of malaria prevention, control and elimination strategies benefiting the entire population in Chad.

The study recommends that the MOH and the NMCP organize community education and sensitization programs within nomadic communities, especially those in remote rural areas, emphasizing the effects of malaria. The NMCP should also enhance both the coverage and use of LLINs and SMC, along with promoting of treatment-seeking behaviors in nomadic communities.

### Electronic supplementary material

Below is the link to the electronic supplementary material.

Supplementary Material 1

Supplementary Material 2

Supplementary Material 3

## Data Availability

The datasets generated and/or analyzed during the current study are available from the corresponding author on reasonable request.

## Abbreviations

ANC: Antenatal care
AOR: Adjusted odd ratio
COR: Crude odd ratio
DNA: Deoxyribonucleic acid
IPTp: Intermittent preventive treatment for pregnant women
LLIN: Long-lasting insecticide-treated net
MOH: Ministry of Public Health
NMCP (PNLP): National Malaria Control Program (*Programme National de Lutte contre le Paludisme*)
PCR: Polymerase chain reaction
SMC: Seasonal malaria chemoprevention
SPAQ: Sulphadoxine-pyrimethamine and amodiaquine
RDT: Rapid diagnostic test
UNICEF: United Nations Children’s Fund
WHO: World Health Organization
