# Supplementary material for: Malaria infection and predictor factors among Chadian nomads’ children
Source: BMC Public Health. 2024 Mar 28;24:918. doi: 10.1186/s12889-024-18454-5 (PMC10979592; doi:10.1186/s12889-024-18454-5)
Supplement: Supplementary file 2 — Supplementary Material 2 [file 12889_2024_18454_MOESM2_ESM.docx]

## **Blood collection form**

Research project: Modeling the dynamics of malaria transmission in a Chadian nomadic setting

Principal investigator: xxxxx

Area : **Dourbali/Massenya/Massaguet/Niellim** Group : **Arab/Fulani/Daza**

| Household ID | Sample code | Age (months/years) | Sexe (M/F) | TDR (+/-) | Temperature (°C) |
| --- | --- | --- | --- | --- | --- |
|  | DLIA1 |  |  |  |  |
|  | DLIA2 |  |  |  |  |
|  | DLIA3 |  |  |  |  |
|  | DLIA4 |  |  |  |  |
|  | DLIA5 |  |  |  |  |
